# Protists show high resilience and thrive under multiple chemical stressors

**DOI:** 10.1002/mlf2.70083

**Published:** 2026-06-25

**Authors:** Jijuan Ding, Fei Liu, Yuanchen Zhao, Zhili He, Yijing Shi, Longfei Shu

**Affiliations:** ^1^ School of Environmental Science and Engineering, Guangdong Provincial Key Laboratory of Environmental Pollution Control and Remediation Technology Sun Yat‐sen University Guangzhou China; ^2^ Marine Synthetic Ecology Research Center Southern Marine Science and Engineering Guangdong Laboratory (Zhuhai) Zhuhai China; ^3^ SCNU Environmental Research Institute, School of Environment, Guangdong Provincial Key Laboratory of Chemical Pollution and Environmental Safety & MOE Key Laboratory of Theoretical Chemistry of Environment South China Normal University Guangzhou China

## Abstract

Protists are an underexplored but functionally important component of aerobic‐activated granular sludge under pollution stress. Using metagenomics, we profiled protistan responses to ciprofloxacin, triclosan, and Cu^2+^ (alone or in combination). Protists remained a stable 6.35%–7.88% of the bacterial community, and the consumers were the most abundant groups. Ciprofloxacin showed little effect on protist abundance, while Cu^2+^ increased protist abundance, especially consumers. Stress conditions also strengthened predominantly positive protist–bacteria associations, suggesting cross‐domain interactions that may enhance community resilience. These results demonstrate that protists are key determinants in stabilizing microbial communities under multiple stressors.

Biological treatment is an effective approach for wastewater treatment, in which microbial communities drive organic matter degradation, nitrogen transformation, and overall system stability. However, microbial community stability can be disrupted by environmental stressors, and different stressors can trigger distinct microbial responses. For example, the extensive use of synthetic antibiotics such as ciprofloxacin (CFX) can reduce microbial diversity and increase the abundance of antibiotic‐resistance genes^1^. While bacteria are the most frequently studied members of microbial communities under treatments, micro‐eukaryotes such as protists are also important and may show greater resilience than bacteria to certain exogenous stressors^2^. This indicates a need to better understand the structure and stress responses of protistan communities.

Protists are diverse unicellular eukaryotes (excluding plants, fungi, and animals) that contribute substantially to microbial community function^3^. Based on ecological roles, protists can be grouped into phototrophs, consumers, and parasites^4^. Phototrophs (e.g., diatoms) support primary production. Consumers influence energy flow and element cycling through predation and interactions with other microorganisms. Parasites include taxa that infect hosts and may live as parasitic symbionts with animals, plants, or other protists. Protists are also used in various biotransformation processes, including the production of specific products^5^. However, how these functional groups are distributed and how they shift in biological treatment systems, especially under stress, remains unclear.

Microbial eukaryotes play key roles in wastewater treatment despite being less abundant than bacteria^6^. Nevertheless, protistan phenotypes and community‐wide patterns under stressful conditions are still insufficiently characterized. In practice, antibiotics, sterilants, and heavy metals commonly co‐occur in wastewater and can substantially affect microbial communities in aerobic‐activated granular sludge. Unlike antibiotics and disinfectants, heavy metals are not degradable and can impose persistent, multi‐target stress on microbial communities^7^. CFX is widely used and environmentally persistent with broad‐spectrum antibacterial activity^1^. Triclosan (TCS), a common sterilant, is a synthetic antimicrobial agent that is toxic to microbes and aquatic organisms, and can decrease bacterial diversity and abundance in activated sludge^8^. Under Cu²⁺ stress, protists may show dual responses: Low concentrations can enhance protistan predation on bacteria^9^, whereas higher concentrations can be toxic. This dose‐dependent behavior suggests that protistan responses may depend not only on stressor identity but also on concentration and co‐stressor context. Because protistan diversity and community structure vary with environmental conditions, protists may also indicate ecosystem health.

Although microbial communities underpin biological treatment performance, their responses under combined stressors remain incompletely understood, particularly for protists. Most previous work has focused on single stressors and emphasized microbial function rather than community dynamics, especially among eukaryotes. Given close protist–bacteria interactions, including the possibility that protists serve as reservoirs for bacterial pathogens^10^, protists may respond indirectly to pollutants through altered interactions. Therefore, this study investigates how protistan communities and their interactions with bacteria change under multiple stressors.

Based on differences in stressor mechanisms, we hypothesize that CFX has limited direct impact on protists, low Cu²⁺ may stimulate protists (e.g., amoebae), and broad‐spectrum antimicrobials may inhibit protists. We designed experiments using CFX as a baseline stressor and superimposed Cu²⁺ and TCS in different combinations. Four aerobic granular sludge bioreactors (R_1_–R_4_) were constructed and exposed to these stressor combinations (Figure 1A). The results provide new insights into protistan responses under multiple chemical stressors and their interactions with bacterial communities in aerobic granular sludge bioreactors.

Bacteria dominated (about 90%), followed by eukaryotes (4.13%–10.34% within all samples), while archaea and viruses each contributed <1% (Figure S1A, within all samples, R_0_–R_4_). Within eukaryotes, protists accounted for 6.35%–7.88% across treatments (Figures 1B and S1B, within all samples, R_0_–R_4_). R_2_ (CFX + Cu²⁺) showed the highest protistan abundance, and both R_2_ and R_4_ (CFX + Cu²⁺ + TCS) showed significantly higher protistan composition than R_0_ (initial), R_1_ (CFX), and R_3_ (CFX + TCS) (Figure S1C, *p* < 0.05), suggesting that Cu²⁺ promoted protistan enrichment.

To contextualize these results, we compared them with protistan profiles from wastewater treatment plant (WWTP) datasets. Eukaryotic abundance in WWTPs ranged from 0.81% to 2.39%, significantly lower than in this study (Figure S2A, *p *< 0.05), and protistan composition also differed among WWTPs and our bioreactors (Figure S2B, *p* < 0.05). These results confirm that eukaryotes, including protists, are meaningful components of wastewater microbiomes and may serve as biological indicators of treatment processes. Given these differences, we next examined how each stressor combination altered protistan abundance and composition within our controlled reactors.

Consistent with this interpretation, protistan abundance in R_1_ did not differ significantly from R_0_, and a similar pattern was observed in R_3_, implying that CFX exerted negligible effects on protists at the tested concentration. In contrast, Cu²⁺ increased protistan abundance (R_2_/R_4_ vs. R_0_/R_1_/R_3_), whereas TCS alone did not significantly change total protistan abundance (R_3_ vs. R_0_/R_1_), although protists decreased in R_4_ compared with R_2_, indicating a potential suppressive trend by TCS when combined with Cu²⁺. Dominant taxa differed across treatments (Figure S3): *Kinetoplastea* dominated in R_0_/R_1_/R_3_, while *Echinamoebida* and *Conoidasida* were most abundant in R_2_ and R_4_, respectively (Figure S1D), demonstrating chemical‐specific shifts in protistan composition.

Community‐level ordination further supported these patterns. Principal component analysis (PCA) separated R_2_ and R_4_ from other groups, whereas R_1_ and R_3_ clustered near R_0_ (Figure S1E). Protistan richness ranged from 214 to 375 species, with a significant reduction observed only in R_4_ (Figure S4, ANOVA, *p* < 0.05). Simpson diversity was the highest in R_2_, followed by R_1_ (Figure S1F, ANOVA, *p* < 0.05), indicating enrichment of dominant protists, particularly under Cu²⁺ exposure (e.g., *Echinamoebida* in R_2_; Figure S5, *p* < 0.05). Collectively, these results indicated that CFX and TCS did not strongly alter protist diversity at the tested levels, whereas Cu²⁺ promoted protistan enrichment. TCS likely induced a weak inhibitory trend consistent with its antimicrobial properties. In contrast to protists, bacterial diversity declined under stress (Figure S6). The lowest bacterial richness and Shannon index occurred in R_4_ under combined stressors, indicating that bacteria were more sensitive than protists to multi‐stressor exposure in these systems.

Consumers were the most abundant functional groups in all treatments (ranging from 43.14% to 69.55%), followed by parasites (17.94% to 31.16%) and phototrophs (11.66% to 29.08%) (Figures 1C and S7A). R_2_ showed the highest consumer abundance, accompanied by lower parasite and phototroph proportions relative to other treatments including R_0_ (*p* < 0.05). R_4_ also showed increased consumers compared with R_0_/R_1_/R_3_ (*p* < 0.05), together with increased parasites and decreased phototrophs.

Correlation analysis showed that total protist abundance was strongly positively associated with consumer abundance (Figure S7B, Pearson *r* = 0.87, *p* < 0.05) and negatively associated with parasites and phototrophs, indicating that protistan enrichment in these reactors was primarily driven by consumers. This consumer dominance aligns with patterns reported for many ecosystems^4^, ^11^, although other habitats (e.g., rainforests and mangrove sediments) can be parasite‐dominated^12^, ^13^. Because consumers such as free‐living amoebae have been used as indicators of environmental disturbance (e.g., road salt contamination in lakes)^14^, shifts in protistan functional composition may reflect broader microbial and ecosystem changes^15^.

PCA based on each functional group again separated R_2_ and R_4_ from R_0_, while R_1_ and R_3_ grouped close to R_0_ (Figure S7C–E). Across functional groups, species numbers decreased significantly in R_4_ (Figure S8, *p* < 0.05). Consumers showed higher variability across treatments, whereas parasites and phototrophs were relatively similar among R_0_–R_3_. In addition, similar patterns were observed for other diversity indices. Simpson diversity suggested enrichment of dominant consumer taxa in R_2_ and, to a lesser extent, R_4_. Consumer relative abundance correlated with Simpson diversity (Pearson *r* = 0.91, *p* < 0.001), indicating dominance by a subset of abundant consumer taxa. Phototroph relative abundance correlated with the Shannon index (Pearson *r* = –0.95, *p* < 0.05), and parasite abundance correlated with all three indices (*p* < 0.05), highlighting that different functional groups respond differently to stress. Overall, Cu²⁺ tended to enrich consumers (e.g., *Echinamoebida*), consistent with prior findings that Cu²⁺ can enhance protistan predation^9^, while the reduced protist abundance in R_4_ relative to R_2_ suggests that TCS may counteract this stimulatory effect. These observations support that protists respond actively to chemical stress, and their functional diversity underpins divergent response trajectories.

To evaluate potential inter‐domain interactions, we focused on bacteria–protist networks, because archaea, viruses, and fungi each contributed <1%. Only taxa with total read counts >100 were included (Figure 1D). The global network comprised 4855 bacterial species and 295 protistan species, yielding 101,522 bacteria–protist associations: 91,527 positive and 9995 negative (Figures 1E and S9A; Pearson |*r* | > 0.8, *p* < 0.001). Keystone taxa, which disproportionately influence community structure regardless of their abundance^16^, also participated extensively in these interactions (e.g., *Methyloradius palustris* with 59 protist links and *Plasmodium berghei* with 1322 bacterial links), indicating complex involvement of protists within the broader microbial community. When networks were constructed for each treatment separately, interaction structures differed markedly (Figure S10). Under initial conditions, 4119 protist–bacteria interactions were detected, with 54.79% positive links (Table S2). In all stressed reactors, the proportion of positive interactions increased: >99% in R_1_ and R_3_, and 68.73% in R_2_ and R_4_. Keystone taxa in each treatment formed predominantly positive associations with bacteria: *Rostrostelium ellipticum* in R_1_ connected to 266 bacteria (all positive); *Saprolegnia diclina* in R_2_ linked to 79 bacteria (70 positive); *Plasmodium coatneyi* in R_3_ linked to 578 bacteria (all positive); and *Phytophthora parasitica* in R_4_ showed 110 links with bacteria (104 positive). These results suggest that stress conditions, particularly CFX and TCS, are associated with increased positive connectivity, potentially stabilizing community structure.

The main difference between R_1_/R_3_ and R_2_/R_4_ was the addition of Cu²⁺, which also significantly increased consumers. Positive microbial interactions, such as substrate exchange between algae and bacteria, can enhance community resistance under pollutant stress^17^, and protists can act as environmental reservoirs for bacteria^18^. Thus, enhanced positive associations may contribute to stress tolerance. However, consumer–bacteria predation is typically considered a negative interaction. The enrichment of consumers in R_2_ and R_4_ may therefore explain why their positive‐link proportions were lower than in R_1_ and R_3_, yet still higher than in R_0_. Overall, protists and bacteria tended to form more positive associations under pollutant stress, suggesting interaction restructuring that may buffer ecosystem disturbance.

We further assessed links between protists and bacterial resistance genes relevant to the tested stressors (antibiotic‐, Cu²⁺‐, and TCS‐resistance genes; Table S3). Antibiotic‐ and TCS‐resistance genes were positively related to parasite abundance and negatively related to consumers (Figure S9B,C, *p* < 0.05), whereas Cu²⁺‐resistance genes were positively associated with phototroph abundance (Figure S9D, *p* < 0.05). These patterns imply that protistan functional composition correlates with resistance gene enrichment in bacteria. Unlike some reports suggesting that consumers can increase antibiotics resistance gene (ARG) abundance in soils^19^, our results indicated that parasites and phototrophs were more strongly associated with resistance gene increase, while consumers showed negative associations, possibly due to predation on resistant bacteria. Collectively, protistan functional diversity appears to shape microbial networks and resistance gene profiles under multi‐stressor exposure.

Overall, our results demonstrate that protists are an integral component of aerobic‐activated granular sludge microbiomes and show stressor‐specific shifts in abundance, functional composition, and interaction patterns with bacteria under combined chemical exposure. Cu²⁺ consistently enriches consumer protists, whereas TCS tends to attenuate this enrichment when co‐occurring with Cu²⁺.

**Figure 1:**
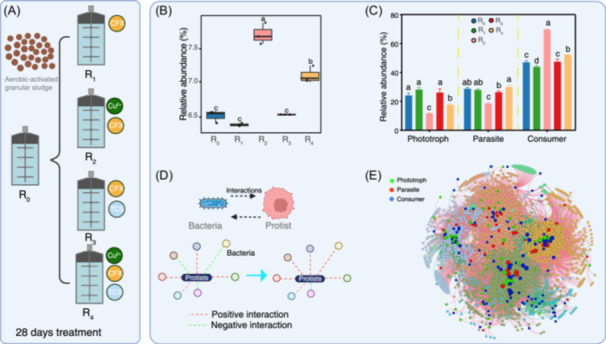
The protist community exhibits adaptability under stressed aerobic‐activated granular sludge reactors and tends to form positive interactions with the bacterial community. (A) Experimental design of this study. The treatment without additional initial stress was set as the control (R_0_). With ciprofloxacin (CFX) as the baseline treatment (R_1_), the combined stress effects of CFX together with Cu²⁺ and triclosan (TCS) on protist communities (R_2_, R_3_, and R_4_) were investigated. (B) The boxplot showing the changes of protists in the eukaryotic communities between these bioreactors. (C) The bar chart showing the relative abundance of the three functional communities, with colors representing different treatments. ANOVA analysis and Tukey HSD multiple comparisons were performaed. The different lowercase letters represent significant differences between groups (*p* < 0.05), while the same lowercase letter represents no significant difference. (D) The protistan and bacterial communities tends to establish positive interactions across all treatment groups in this study. (E) The interaction network between protists and bacteria. Each point is a species. The blue points are the consumers, the green points represent the phototrophs, the red points represent the parasites, and the other colored points represent the bacterial species belonging to different separated communities. The size of a point is proportional to the in‐degree. The red lines represent the positive interactions, and the green lines represent the negative interactions. Pearson correlation was used in this network. Only the Pearson |*r*| > 0.8 and adjusted *p* < 0.001 of the edges are shown.

A key limitation is that functional inference and quantitative profiling of protistan genes from metagenomic data remain challenging, partly due to incomplete eukaryotic reference databases and annotation uncertainty. In addition, because our design emphasizes combined‐stressor scenarios, future factorial experiments incorporating single‐stressor gradients are needed to disentangle potential synergistic or antagonistic effects, particularly between Cu²⁺ and TCS.

Finally, the observed links between protistan functional groups and bacterial resistance genes suggest that protistan community structure may provide an ecological context influencing resistance maintenance and potentially horizontal gene transfer. Mechanistic validation using targeted assays (e.g., metatranscriptomics, qPCR of eukaryotic markers, and controlled co‐culture or microcosm experiments) will be critical to resolve causality. Together, these findings broaden our understanding of wastewater microbiomes by highlighting protists as active participants in both biotransformation processes and stress responses.

## ETHICS STATEMENT

No human or animal subjects were used in this study.

## Supporting information

Supplementary information.

## Data Availability

All of the metagenomic sequencing reads were submitted to the National Center for Biotechnology Information Short Reads Archive (NCBI SRA) database under the project PRJNA1049193. The materials and methods are provided in the Supporting Information.
