# Supplementary material for: Protists show high resilience and thrive under multiple chemical stressors
Source: mLife. 2026 Jun 25;5(3):388–92. doi: 10.1002/mlf2.70083 (PMC13327597; doi:10.1002/mlf2.70083)
Supplement: Supplementary file 1 — Supplementary information. [file MLF2-5-388-s001.docx]

*Supplementary information for*

**Protists exhibit high resilience and thrive under multiple chemical stressors**

Jijuan Ding^a^, Fei Liu^a^, Yuanchen Zhao^a^, Zhili He^c^, Yijing Shi^b*^, Longfei Shu^a*^

^a^School of Environmental Science and Engineering, Southern Marine Science and Engineering Guangdong Laboratory (Zhuhai), Guangdong Provincial Key Laboratory of Environmental Pollution Control and Remediation Technology, Sun Yat-sen University, Guangzhou 510006, China

^b^SCNU Environmental Research Institute, School of Environment, Guangdong Provincial Key Laboratory of Chemical Pollution and Environmental Safety & MOE Key Laboratory of Theoretical Chemistry of Environment, South China Normal University, Guangzhou, 510006, China

^c^Marine Synthetic Ecology Research Center, Southern Marine Science and Engineering Guangdong Laboratory (Zhuhai), Zhuhai 509082, China

**Corresponding authors:**

Prof. Longfei Shu

School of Environmental Science and Engineering, Sun Yat-sen University, Guangzhou 510006, China

Email: [shulf@mail.sysu.edu.cn](mailto:shulf@mail.sysu.edu.cn)

Or Prof. Yijing Shi

School of Environment, Guangdong, South China Normal University, Guangzhou, 510006, China

Email: [yijing.shi@m.scnu.edu.cn](mailto:yijing.shi@m.scnu.edu.cn)

## Introduction

Experimental Design and Plan

Previous studies have found that the efficiency of aerobic granular sludge reactors can be changed due to pressure, and this process is usually caused by changes in the microbial community [1]. Pollutants are known to alter bacterial community structures by affecting interaction dynamics [2]. The researchers have found the effects of stress to the microbial functions but ignored the changes of the microbial communities, especially the eukaryotes. Given the close interaction between protists and bacteria, with protists potentially serving as reservoirs of bacterial pathogens within environments [3], it is reasonable to hypothesize that protists would also experience changes in response to stressors, albeit indirectly. Previous studies mostly focus on single stress and ignore the response mechanism of protists under combined stress. We seek to understand how interactions between protists and bacterial communities change under the stress of multiple pollutants, such as the influence of protistan predation on bacteria or bacterial reservoirs harbored by protists.

In general, due to the differences in the mechanisms of antibiotics, this study holds that bacterial antibiotics do not have a significant impact on protists; however, low concentrations of Cu²⁺ have a stimulatory effect on protists (such as amoebae); and broad-spectrum synthetic antimicrobial agent also have an inhibitory effect on protists. Based on this, this study designed an experiment that uses bacterial antibiotics as the substrate and superimposes other potential stress factors to test the response of protist communities to combined stresses. This study additionally selected a Cu^2+^ concentration that is potentially beneficial to protists and a factor that may have a negative impact on protists; through different combinations of these factors, we aim to reveal the response of protist communities under the stress of multiple chemical factors. Specifically, this study selected ciprofloxacin (CFX), triclosan (TCS), and copper sulfate (Cu^2+^) as representative chemicals. These chemicals are widely reported in the environment and are directly related to microbes. Four aerobic granular sludge bioreactors (R_1_–R_4_) were constructed and exposed to different combinations of these stressors. These findings provide novel insights into the behavior of protistan communities under multiple stressors and their interactions with bacterial communities, deepening our understanding of microbial dynamics in aerobic granular sludge bioreactors.

## Result and discussion

**The functional potential of protists in response to chemical stressors**

To investigate the functions within the protistan contigs, we predicted the potential genes within them. There were 1,449 predicted genes annotated into 173 pathways by KEGG serves, ranging from 37 to 1,064 within all aerobic granular sludge bioreactors (Figure 5A, Figure S8). The most common genes were RAC1 (K04392, 69 predicted genes), which was related to the MAPK signaling pathway. These genes are closely related to cell movement, and the significant increase in the number of these genes might be related to the higher mobility of protists ^[25]^. The most abundant pathway was oxidative phosphorylation, which counted for 414 predicted genes, followed by ribosome-related genes, that are directly related to energy acquisition and element transformation in protists. In addition, there were more predicted genes in R_4_, such as transporters. We found that the R_4_ showed the most abundant predicted genes (1,055.67 on average) in all aerobic granular sludge bioreactors (Figure S9, ANOVA, *p* < 0.05), followed by R_2_, and the predicted genes did not show significant differences between the R_0_, R_1_, R_3_ (ANOVA, *p* > 0.05). The number of predicted genes was proportional to the proportion of protists in these aerobic granular sludge bioreactors. Our results showed that there were abundant protistan functional genes in these treatments. As the relative abundance of protists increased, so did the diversity of predicted genes in these protistan communities.

When we transformed the counts of predicted genes into relative abundance (Figure 5B), we found that the oxidative phosphorylation and ribosome-related genes were still the most abundant genes in these treatments. However, their relative abundance was decreased in R_2_ and R_4_, and more other predicted genes were found in R_4_, such as cofactor-related genes. The other parts of the predicted genes showed higher percentages in R_2_ and R_4_ than others (ANOVA, *p* < 0.05), such as signaling protein. In addition, there was no significant difference between R_1_ and R_3_ (ANOVA, *p* > 0.05), which indicated that their functional structure was similar. These results showed that the functional gene structures of protists were significantly affected by different treatments in these aerobic granular sludge bioreactors, resulting in significant differences in the abundance of functional genes. It is mainly shown in the bioreactors with Cu^2+^ added (R_2_ and R_4_), and these increased abundances of consumers were accompanied by multiple predicted genes involved in metabolism-related functions.

Within these predicted genes, the most common genes were involved in movement. The deformation and movement of cells are significantly related to their predatory habits ^[26]^, and they are one of the most primitive connections between protists (or consumers) and bacteria ^[27]^. Our results showed that the increasing relative abundance of consumers was associated with the predatory, and the other evidence was the increasing energy metabolism and material transformation process. Because of the negative interaction between consumers and bacteria ^[20]^, the percentage of positive relationships was decreased in R_2_ and R_4_ compared to R_1_ and R_3_. Our results indicated that multiple factors, including the changes in internal functions and external stressors, influenced the interactions in the microbial communities.

## Material and methods

### Chemicals

The CFX was dissolved in water with 10% acetic acid (50:50 v/v, 100 mg/L). The TCS stock solution was prepared in methanol, and the final concentrations would be 5 g/L. The Cu^2+^ was represented by CuSO_4_ with a stock solution of 17.3 g/L.

### Experiment design

Four parallel aerobic granular sludge reactors were set up in cylindrical sequencing batch reactors (SBRs) with a working volume of 1.4 L (Φ = 70 mm; H = 360 mm). The reactors were operated sequentially with five successive phases (a 6-h cycle). Specifically, 6 min feeding, 30 min anoxic phase, 318 min aeration, 3 min settling, and 3 min withdrawal within one cycle. The volumetric exchange ratio was set at 50%, resulting in a hydraulic retention time of 12 hours. Aerobic granular sludge (average particle size = 1,650 μm, namely R_0_) from a mother SBR operated in our laboratory for over a year was collected and inoculated to the four SBRs, namely R_1_, R_2_, R_3,_ and R_4_, respectively. The synthetic wastewater consisted mainly of sodium acetate (500 mg/L chemical oxygen demand, COD), ammonium chloride (150 mg NH_4_^+^-N/L), and monopotassium phosphate (5 mg P/L). Other necessary nutrients were supplied [4]. After a week stabilization period, chemicals were added to the reactors, with CFX (100 µg/L) only for R_1_, CFX (100 µg/L) and Cu^2+^ (2 mg/L) for R_2_, CFX (100 µg/L) and TCS (100 µg/L) for R_3_, and CFX (100 µg/L), TCS (100 µg/L) and Cu^2+^ (2 mg/L) for R_4_. his Cu^2+^ feed rate was chosen because a previous study identified toxic effects at 10 mg/L Cu^2+^ in bench-scale activated sludge reactors [5], while 2 mg/L Cu^2+^ had no distinct impact on microbial activity [6]. Additionally, the World Health Organization (WHO) has established a provisional health-based guideline value of 2 mg/L for copper in drinking water. It was desired to evaluate the influence of “typical” rather than a priori toxic Cu^2+^ levels. The reactors were exposed to three chemicals for four weeks (28 days). Then, the sludge was taken out for further analysis, and three parallel samples were taken from each reactor.

The DNA was extracted with 5.0 g samples using the methods described previously [7]. A nanodrop ND-2000 Spectrophotometer (Thermo Fisher Scientific, MA, USA) was used to determine the DNA purity and concentrations. DNA fragment libraries were subjected to metagenomic sequencing on Illumina Novaseq PE150 to generate paired-end reads. All of the metagenomic sequencing reads were submitted to the National Center for Biotechnology Information Short Reads Archive (NCBI SRA) database under the project PRJNA1049193 (Table S1).

### The assembly and identification of protists

We used the Trimmomatic (v0.36) [8] to filter the low quality of raw data (parameters: TruSeq3-PE.fa 2:30:10, LEADING 5, TRAILING 5, SLIDINGWINDOW 5:20, and MINLEN 30). The clean data was then assembled by MEGAHIT (default parameters: -k-list 21, 29, 39, 59, 79, 99, 119, and 141) [9] in the MetaWRAP pipeline (v1.3.2) [10], and the contigs less than 1000bp would be removed.

For the clean reads, the Kraken2 (v2.1.0) [11] was used to assign their taxonomy (parameters: -paired -use-names -use-mpa-style -report-zero-counts) using the Kraken database Standard_PlusPF, which contains Standard plus Refeq protozoa & fungi, and the Standard database contain the Refeq archaea, bacteria, viral, plasmid, human, and UniVec_Core. As for the results, a custom Python script was used to filter and classify the results. Then, the count number was transformed to relative abundance. According to the previous study [12, 13], we annotated the protists as three functional groups, including phototrophs, parasites, and consumers.

For the filtered contigs, the potential eukaryotic contigs were reconfirmed and taxonomic assigned using Kaiju (v1.9.2) with the nr_euk database [14]. Then, eukaryotic genes were predicated from eukaryotic contigs using MetaEuk (Release 6-a5d39d9) [15] reference to the UniRef90 database [16]. The predicted gene was annotated using the KEGG server (BlastKOALA) [17, 18]. It is exceeding hard to recover the eukaryotic genomes from metagenomes [19], and there are no suitable tools to calculate the abundance of the predicated eukaryotic genes. Thus, we did not reassemble and evaluate the single genome of protists and only counted the number of them.

### Annotation of resistant genes

For the contigs that were not assigned to eukaryotes, the open reading frames (ORFs) within these contigs were predicted by Prodigal (v2.6.3) [20]. The abundance of each ORFs was calculated by salmon (v1.6.0) [21] using Transcripts per Kilobase per Million mapped reads (TPM). For the ARG annotation, the ORFs were blasted against a structured database of antibiotic resistance genes (SARG, version 3-S) (https://smile.hku.hk/ARGs) [22] by DIAMOND (v2.1.8.162) [23]. The Cu^2+^ and TCS-resistant genes were conducted similarly by searching against the Antibacterial Biocide and Metal Resistance Genes Database (BacMet, version 2.0, <http://bacmet.biomedicine.gu.se>) [24]. The three resistant genes were filtered with a threshold of *E*-value ≤ 10^–5^, query coverage ≥ 90%, and ≥ 50% amino acid identity.

### Statistical analysis

All statistical analyses were conducted using R (v4.0.5) and Python (v3.7.10), along with relevant packages. R and Python were used to perform the Analysis of Variance (ANOVA) test, Tukey's Honest Significant Difference Test (Tukey HSD test), Pearson correlations, and linear regressions. Principal Component Analysis (PCA) was employed using the vegan package. The ComplexHeatmap [25] was used for heatmap visualization. The other methodology for each statistical analysis is described in conjunction with its results. The current studies show that the protists own wide and complex interactions with bacteria [26]. The R packages, psych, and corrr, were used to analyze the Pearson correlations between protists and bacteria or the abundance of ARGs, and the *p* values of the interaction networks were corrected by FDR. Only the R > 0.8 or R < -0.8, and adjusted *p* < 0.01 of the edges were shown. The networks were visualized by Gephi.

The reference WWTP datasets:

Vermont (n=18) [27]


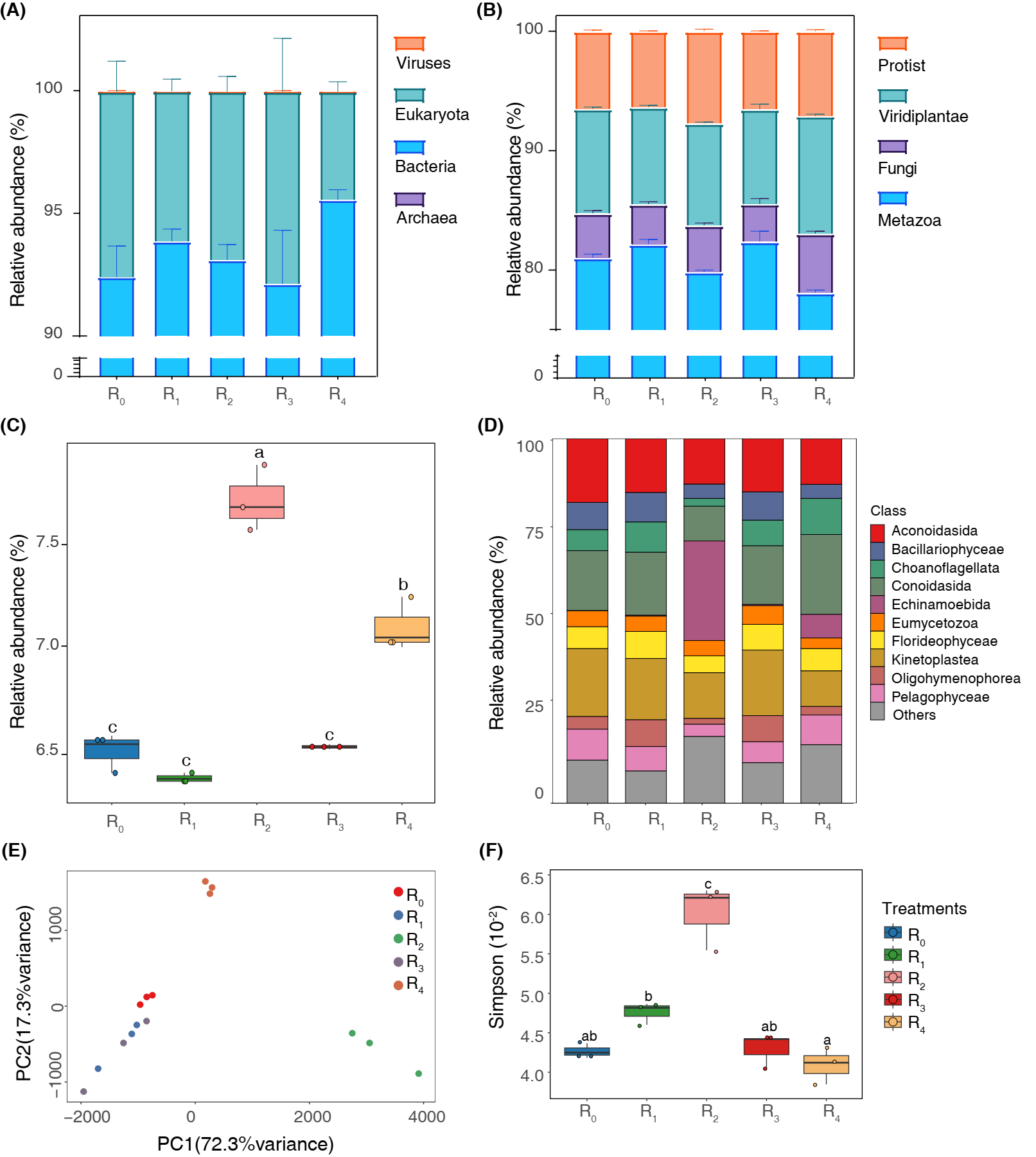


**Figure S1.** The communities in all of four aerobic granular sludge bioreactors. **(A)** The stacked bar plot shows the overall structure of the communities in these bioreactors, including archaea, viruses, eukaryotes, and bacteria. The colors represent their domains. **(B)** The stacked bar plot shows the structure of the eukaryote communities in these bioreactors, including metazoa, viridiplantae, and fungi. The rest of them are protists. The colors represent their kingdoms. **(C):** The boxplot shows the changes of protists in the eukaryotic communities between these bioreactors. **(D)** The stacked bar chart shows the classification of protists at the class level among these bioreactors. **(E)** The scatter plot shows the results of PCA, and the colors represent the different bioreactors. **(F)** The boxplot shows the Simpson index of protists between the bioreactors. In the **(C)** and **(F)**, the differences between bioreactors are marked as the letters (‘a’, ‘b’, and ‘c’); ANOVA analysis showed that there were significant overall differences among the groups (*p* < 0.05), and Tukey HSD multiple comparisons indicated that different letters represent significant differences between groups (*p* < 0.05), while the same letter represents no significant difference.


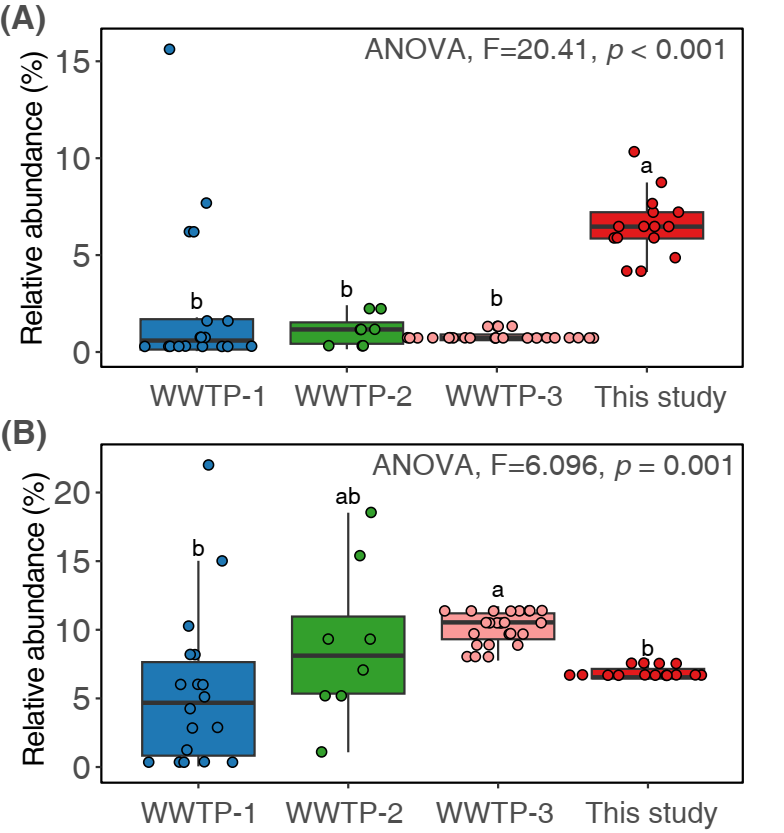


**Figure S2:** (A) The boxplot shows the eukaryotic relative abundance across the three WWTPs and bioreactors in this study. (B) The boxplot shows the protistan relative abundance across the three WWTPs and bioreactors in this study. The differences between bioreactors are marked as the letters (‘a’, ‘b’, and ‘c’); ANOVA analysis showed that there were significant overall differences among the groups (*p* < 0.05), and Turkey HSD multiple comparisons indicated that different letters represent significant differences between groups (*p* < 0.05), while the same letter represents no significant difference.


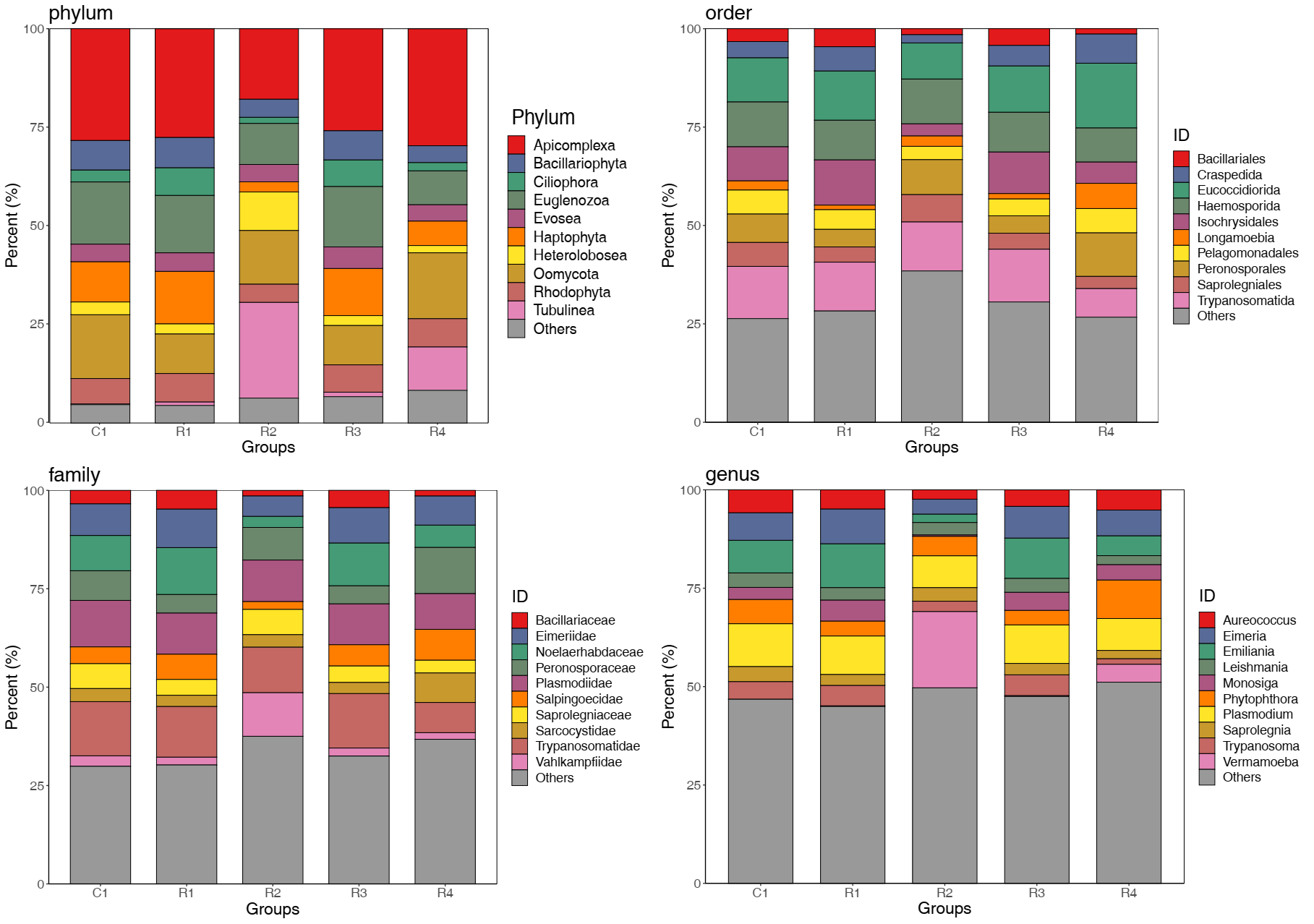


**Figure S3:** The stacked bar chart shows the classification of protists at the four different levels among the treatments.


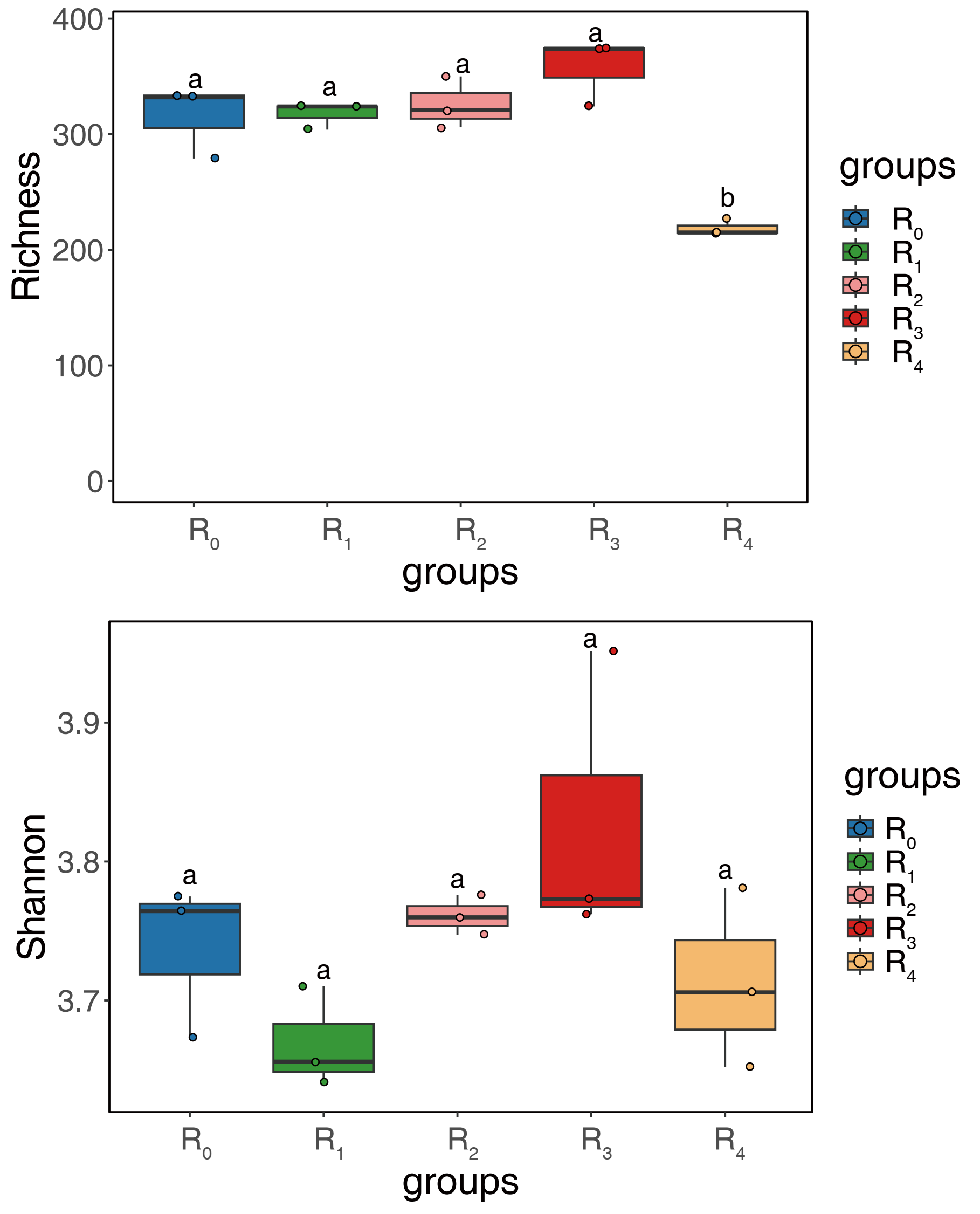


**Figure S4:** Alpha diversity of protistan communities in these treatments. The boxplot of the protistan Richness and Shannon index among them. The differences between bioreactors are marked as the letters (‘a’, ‘b’, and ‘c’); ANOVA analysis showed that there were significant overall differences among the groups (*p* < 0.05), and Turkey HSD multiple comparisons indicated that different letters represent significant differences between groups (*p* < 0.05), while the same letter represents no significant difference.


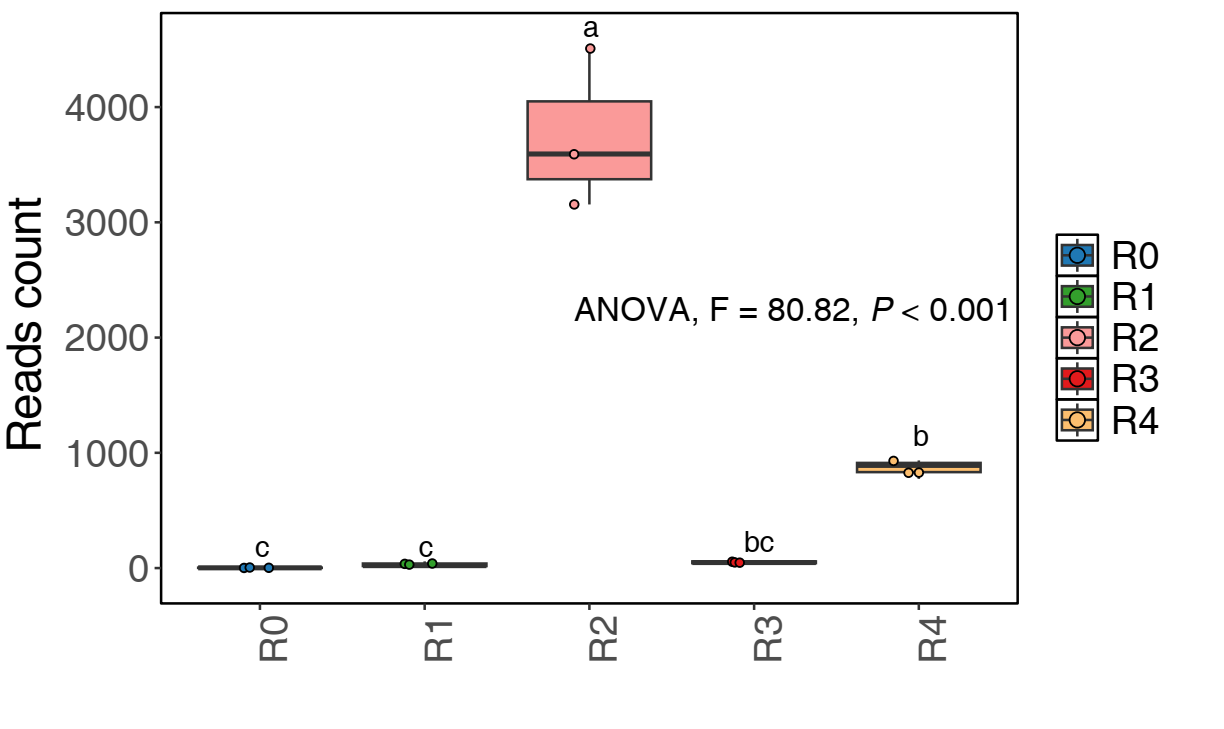


**Figure S5:** The read counts of the *Echinamoebida* in these treatments. The differences between reactors are marked as the letters (‘a’, ‘b’, and ‘c’); ANOVA analysis showed that there were significant overall differences among the groups (*p* < 0.05), and Turkey HSD multiple comparisons indicated that different letters represent significant differences between groups (*p* < 0.05), while the same letter represents no significant difference.


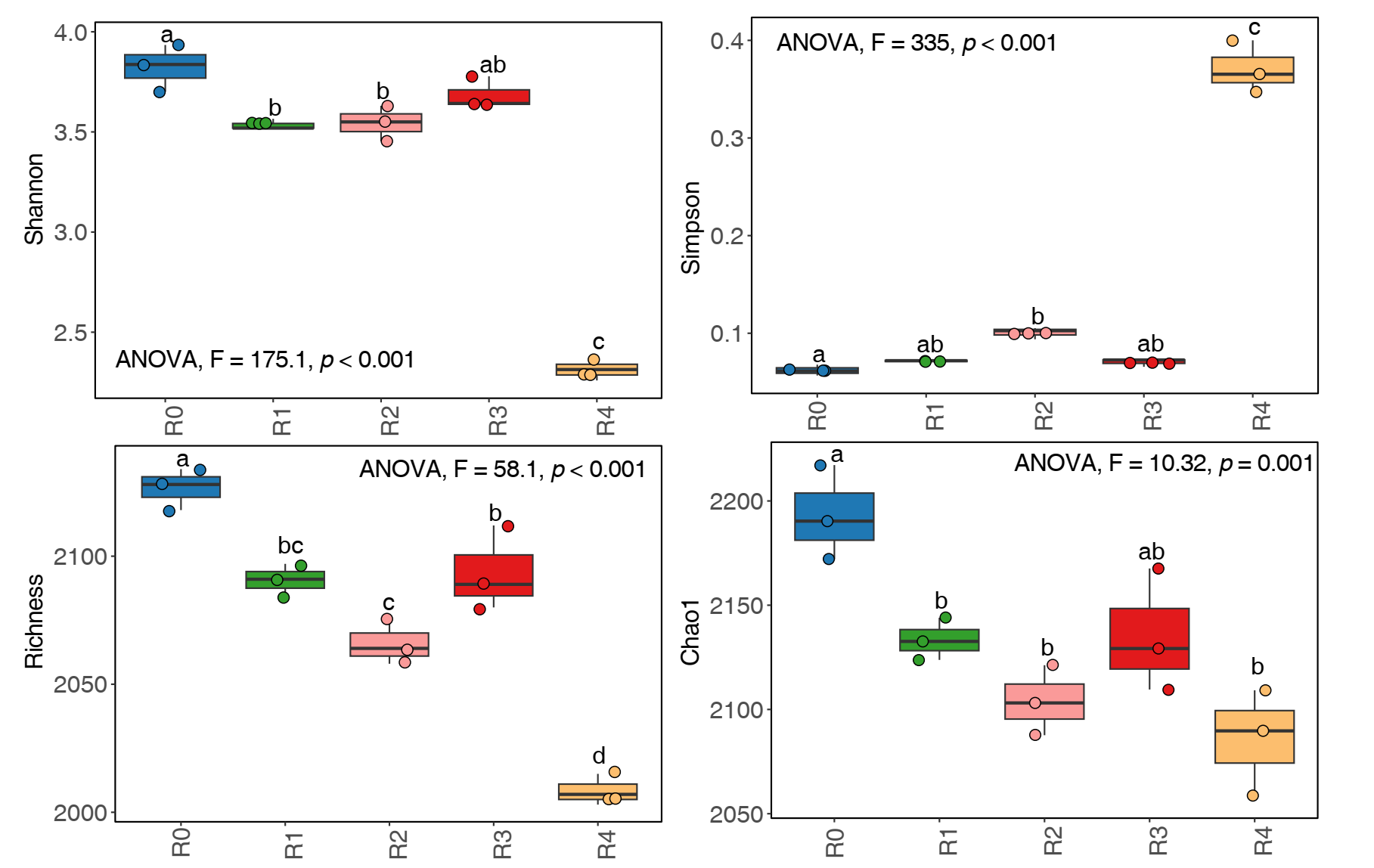


**Figure S6:** Alpha diversity of bacteria communities in these treatments. The boxplot of the bacteria Shannon, Simpson, Richness, and Shannon index among them. The differences between reactors are marked as the letters (‘a’, ‘b’, and ‘c’); ANOVA analysis showed that there were significant overall differences among the groups (*p* < 0.05), and Turkey HSD multiple comparisons indicated that different letters represent significant differences between groups (*p* < 0.05), while the same letter represents no significant difference.


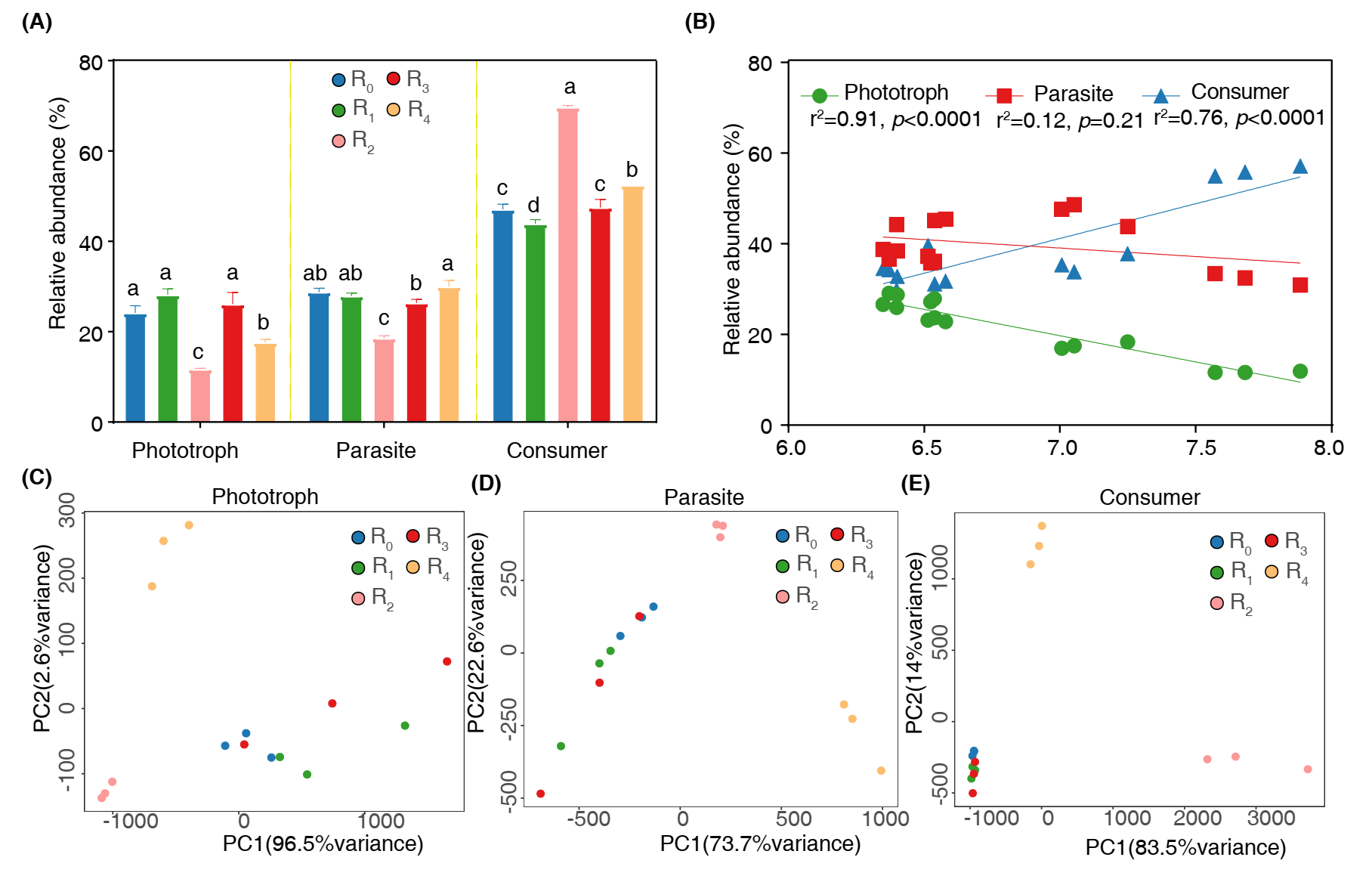


**Figure S7.** The diversity of three protistan functional groups among these four bioreactors. **(A)** The bar chart shows the relative abundance of the three functional communities, with colors representing different treatments. The differences between bioreactors are marked as the letters (‘a’, ‘b’, and ‘c’); ANOVA analysis showed that there were significant overall differences among the groups (*p* < 0.05), and Tukey HSD multiple comparisons indicated that different letters represent significant differences between groups (*p* < 0.05), while the same letter represents no significant difference. **(B)** The relationships of relative abundance between three functional groups (x-axis) and that of protists in microbial communities (y-axis). The line showed the line regression. **(C-E)** The PCA scatter plot shows the cluster of functional groups within different samples, and the colors represent the different bioreactors, including phototroph **(C)**, parasite **(D)**, and consumer **(E)**.


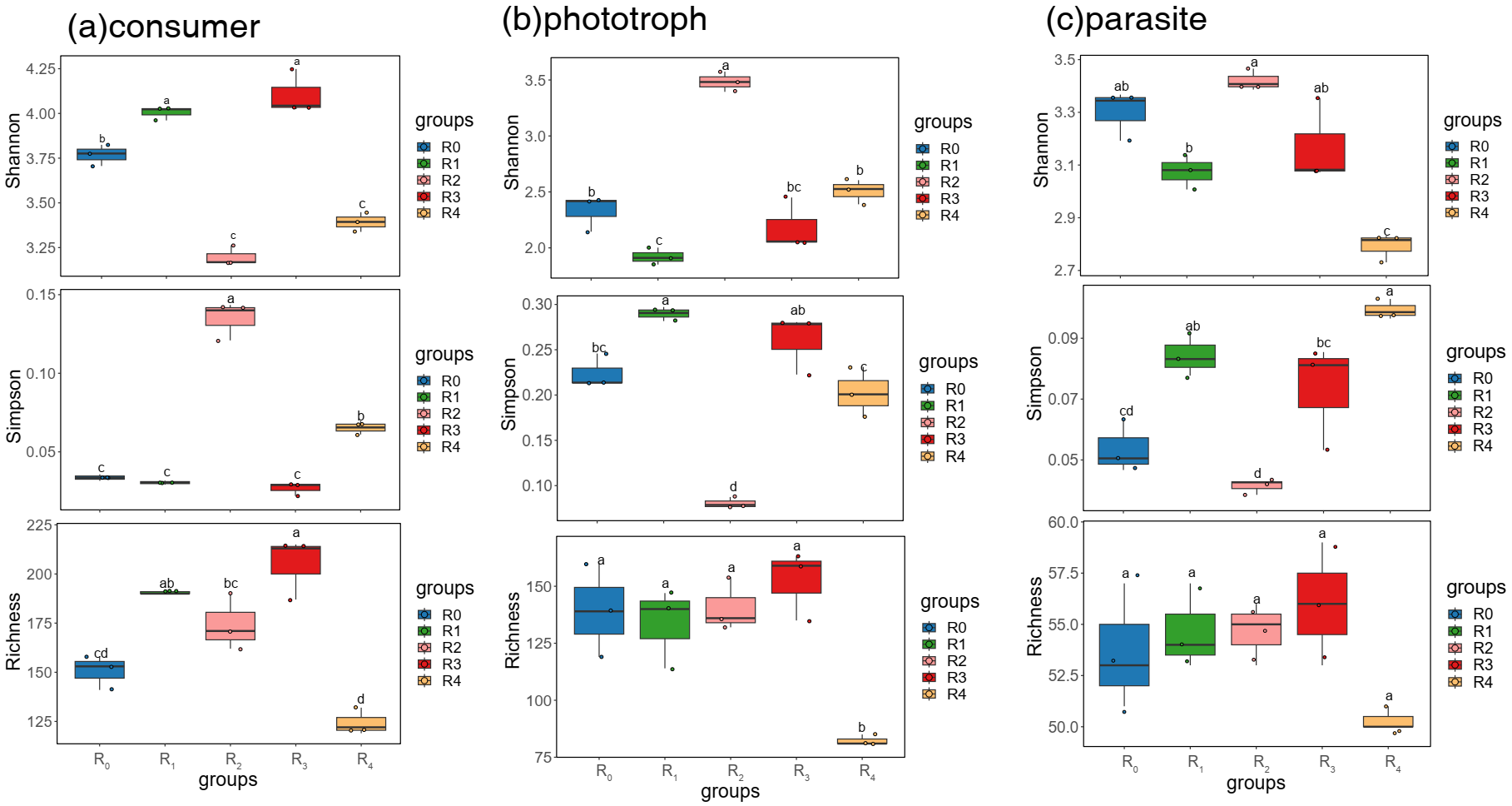


**Figure S8**: Alpha diversity of consumers, phototrophs, and parasites in these treatments. (a): The boxplot of the consumers' Richness, Shannon, and Simpson index among these treatments. (b): The boxplots show the α diversity of phototrophs. (c): The boxplots show the α diversity of parasites. The differences between bioreactors are marked as the letters (‘a’, ‘b’, and ‘c’); ANOVA analysis showed that there were significant overall differences among the groups (*p* < 0.05), and Turkey HSD multiple comparisons indicated that different letters represent significant differences between groups (*p* < 0.05), while the same letter represents no significant difference.


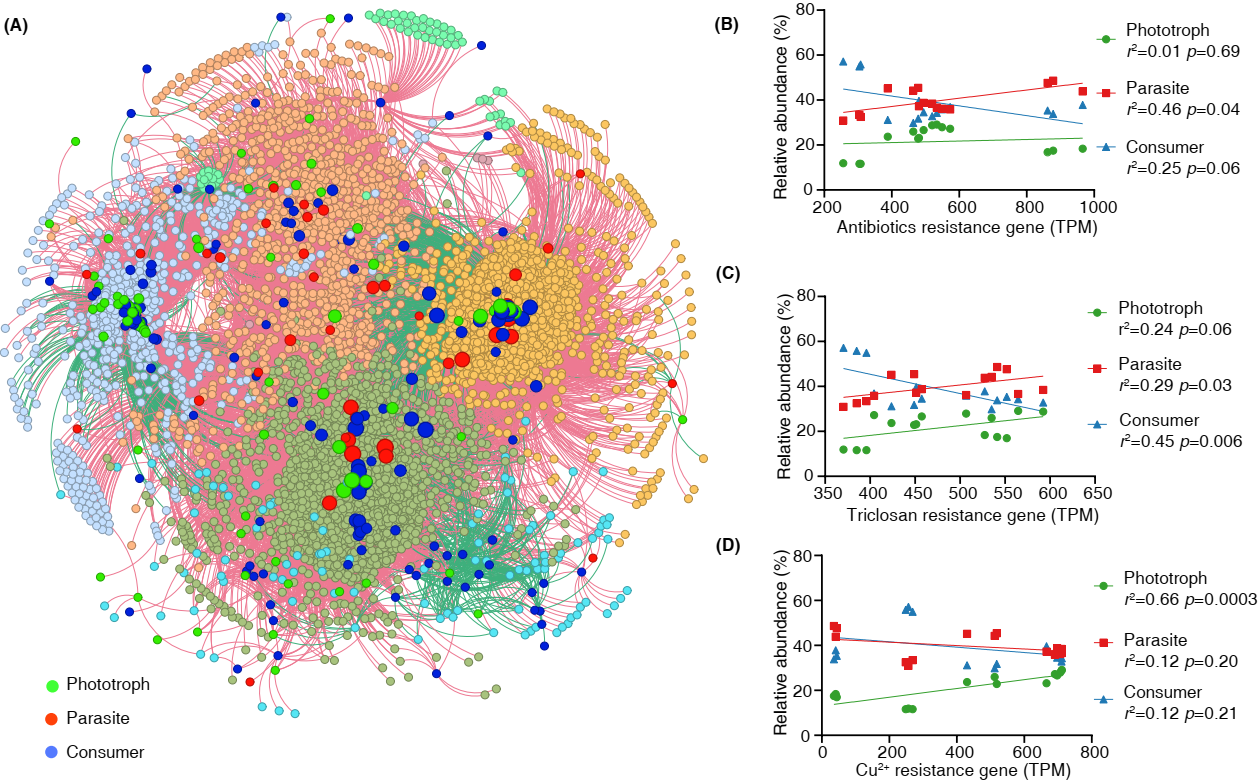


**Figure S9.** Impact of protistan communities on the bacteria. **(A)** The interaction network between protists and bacteria. Each point is a species, and the blue points are the consumers the green points represent the phototrophs, the red points represent the parasites, and the other colored points represent the bacterial species belonging to different separated communities. The size of a point is proportional to the in-degree. The colors of the lines correspond to the type of interaction, while the red lines are the positive interactions, and the green lines are the negative interactions. Pearson correlation is used in this network. Only the R > 0.8 or R < -0.8, and adjusted *p* < 0.01 of the edges are shown. **(B-D)** The scatter plot shows the relationships between the three functional groups (relative abundance) and the abundance of bacteria resistant genes (TPM), including the antibiotics resistance gene (B), Cu^2+^ resistance gene (C), and triclosan resistance gene (D). The line shows the line regression.


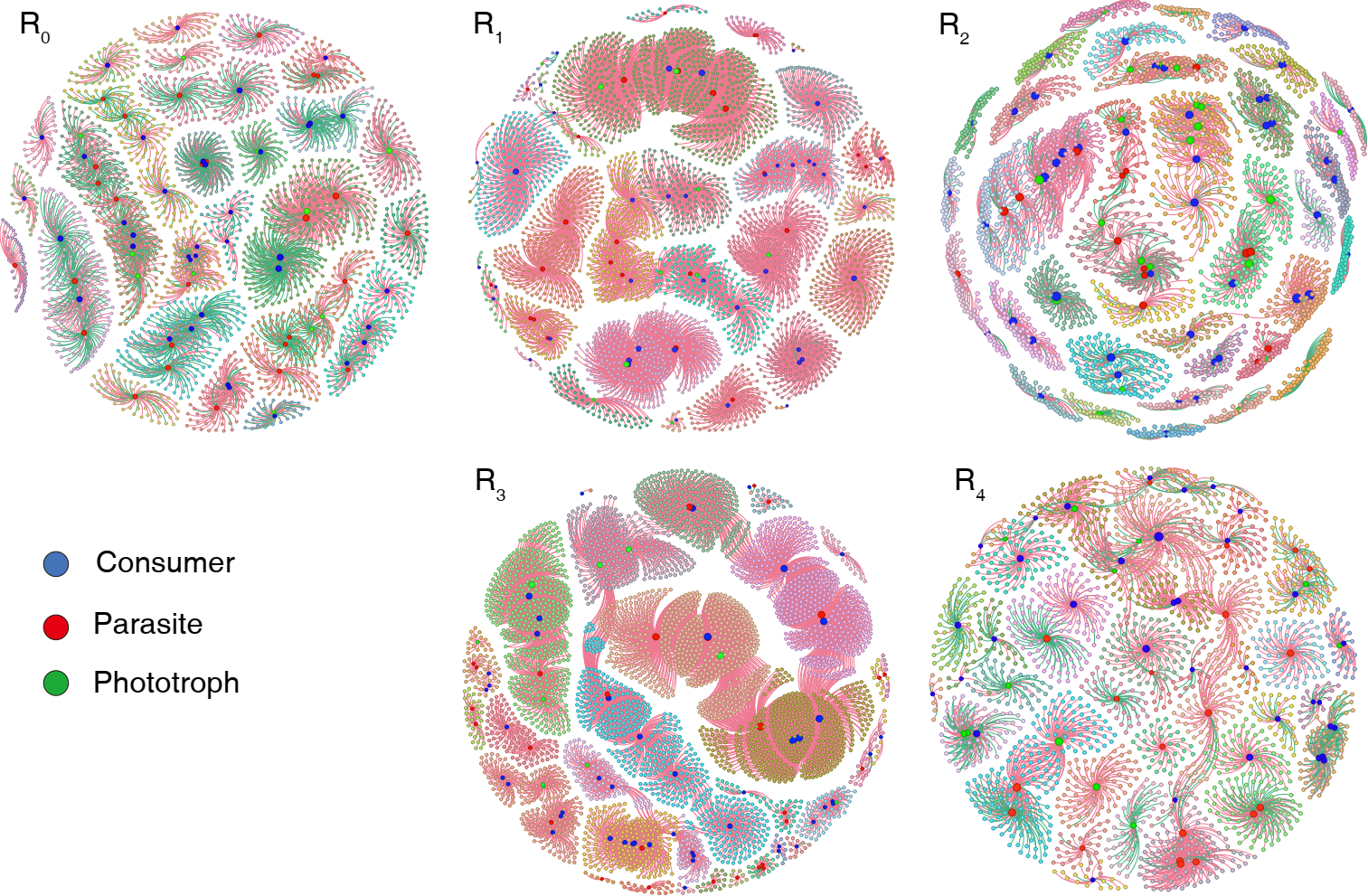


**Figure 10.** The interaction network between protists and bacteria. Each point is a species, and the blue points are the consumers the green points represent the phototrophs, the red points represent the parasites, and the other colored points represent the bacterial species belonging to different communities. The size of a point is proportional to the in-degree. The colors of the lines correspond to the type of interaction, while the red lines are the positive interactions, and the green lines are the negative interactions. Pearson correlation is used in this network. Only the R > 0.8 or R < -0.8, and adjusted *p* < 0.01 of the edges are shown.


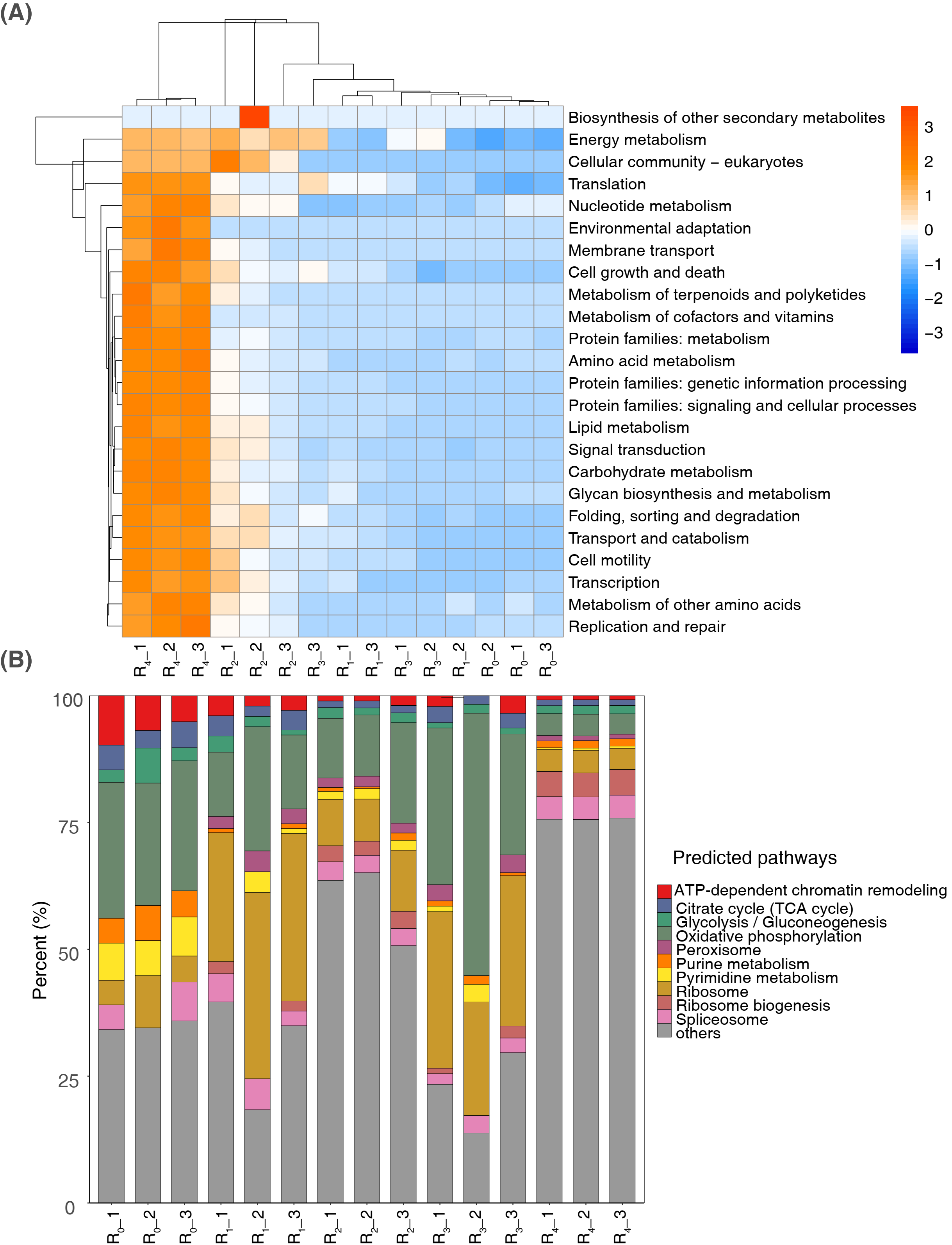


**Figure S11.** The predicated eukaryotic genes in these samples. (A) The functional catalogs among 15 samples. The columns and rows are clustered using the Manhattan and Euclidean distance method, respectively. (B) The composition of eukaryotic genes in these samples. The top 10 genes are shown.


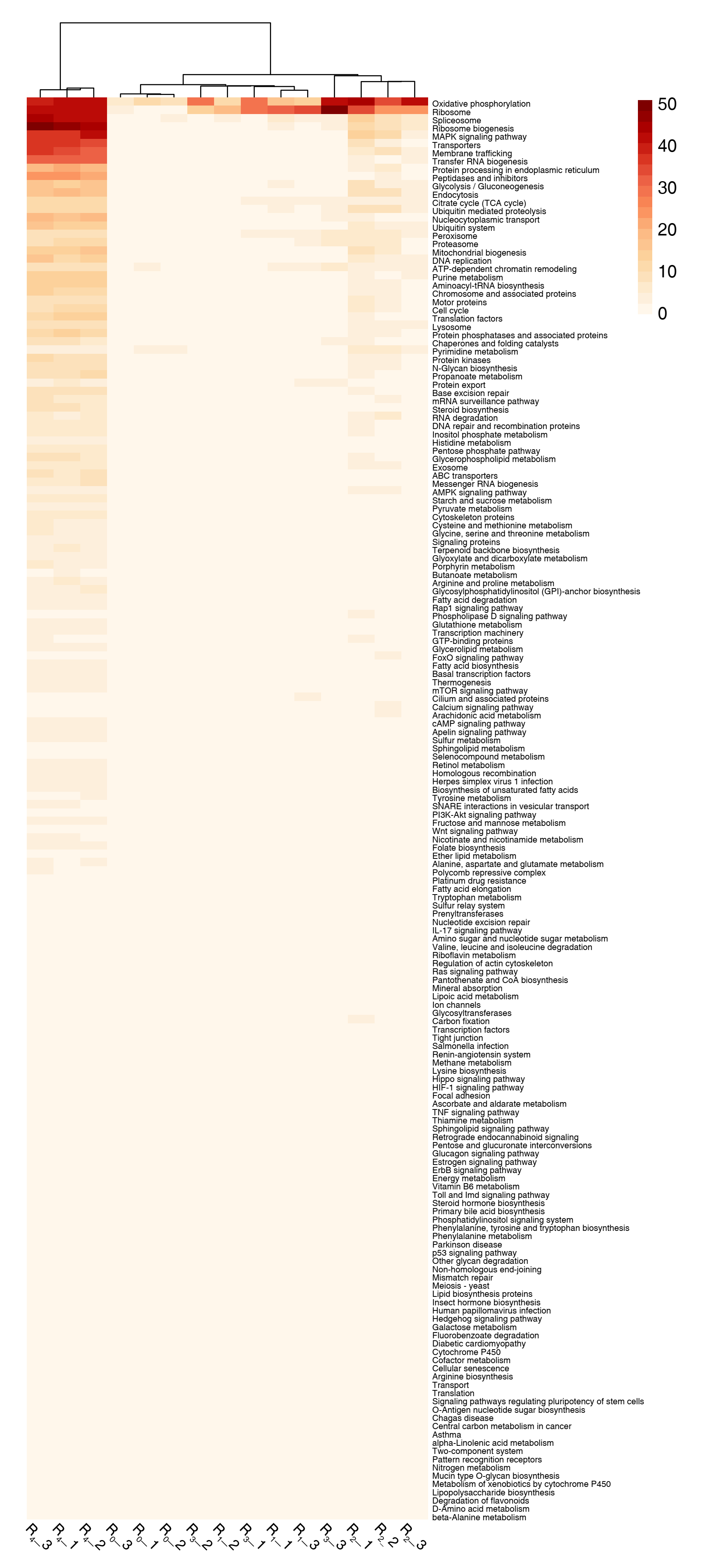


**Figure S12:** The heatmap of the predicted genes among 15 samples. The color represents the amount of the predicted genes. The columns are clustered using the Euclidean distance method.


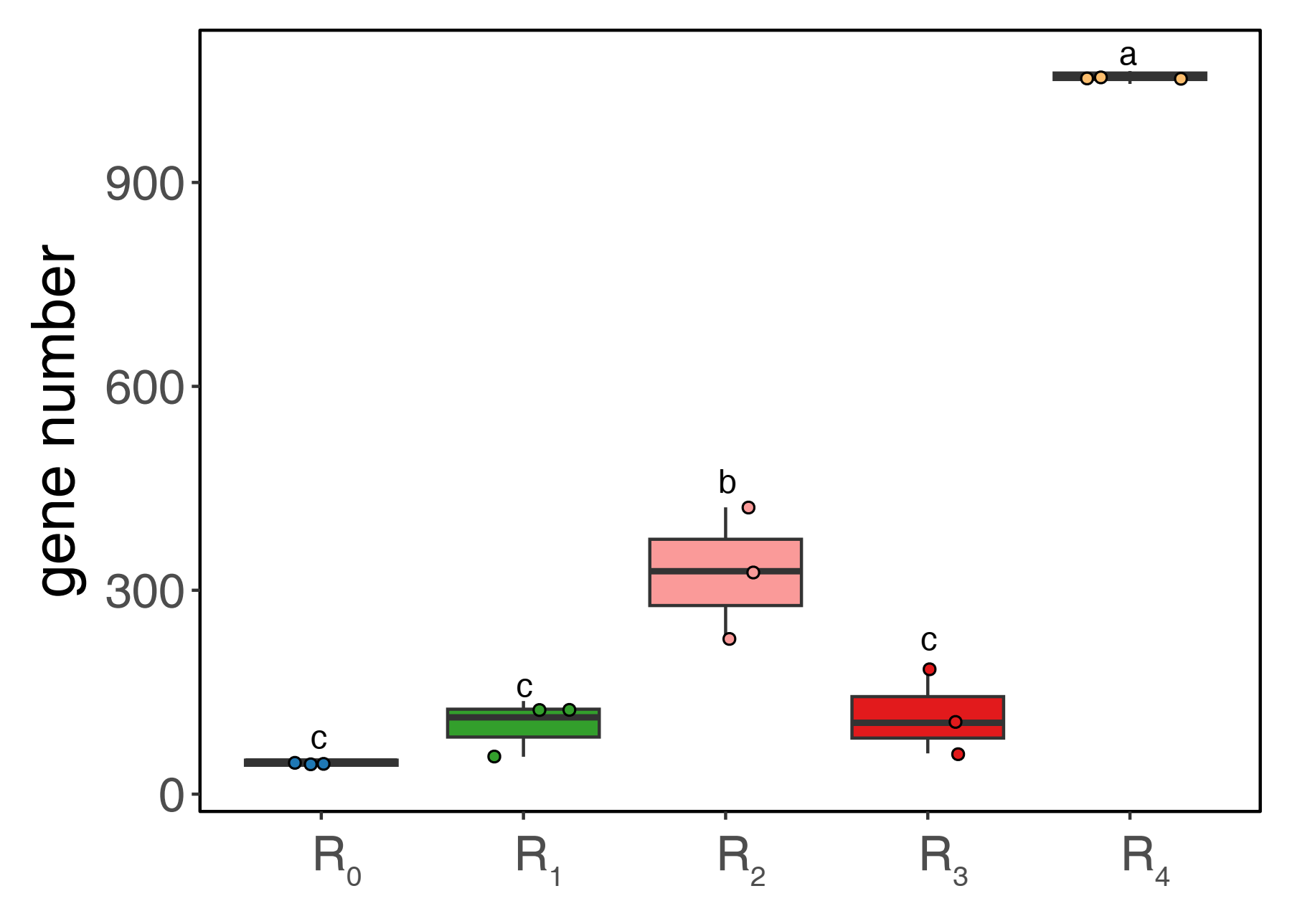


**Figure S13:** The boxplot shows the predicted gene number of protists between treatments. The differences between bioreactors are marked as the letters (‘a’, ‘b’, and ‘c’); ANOVA analysis showed that there were significant overall differences among the groups (*p* < 0.05), and Turkey HSD multiple comparisons indicated that different letters represent significant differences between groups (*p* < 0.05), while the same letter represents no significant difference.

Table S1: Sample information.

| Treatments | Sample ID | Read_id | Size (*GB) |
| --- | --- | --- | --- |
| R_0_ | S1 | S1_1 | 6.17 |
|  |  | S1_2 | 6.15 |
|  | S2 | S2_1 | 7.13 |
|  |  | S2_2 | 7.11 |
|  | S3 | S3_1 | 6.94 |
|  |  | S3_2 | 6.93 |
| R_1_ | S4 | S4_1 | 9.35 |
|  |  | S4_2 | 9.34 |
|  | S5 | S5_1 | 6.68 |
|  |  | S5_2 | 6.67 |
|  | S6 | S6_1 | 6.28 |
|  |  | S6_2 | 6.26 |
| R_2_ | S7 | S7_1 | 5.79 |
|  |  | S7_2 | 5.78 |
|  | S8 | S8_1 | 4.9 |
|  |  | S8_2 | 4.89 |
|  | S9 | S9_1 | 5.29 |
|  |  | S9_2 | 5.27 |
| R_3_ | S10 | S10_1 | 10.46 |
|  |  | S10_2 | 10.44 |
|  | S11 | S11_1 | 7.1 |
|  |  | S11_2 | 7.07 |
|  | S12 | S12_1 | 5.59 |
|  |  | S12_2 | 5.57 |
| R_4_ | S13 | S13_1 | 6.7 |
|  |  | S13_2 | 6.69 |
|  | S14 | S14_1 | 6.98 |
|  |  | S14_2 | 6.97 |
|  | S15 | S15_1 | 8 |
|  |  | S15_2 | 7.98 |

Table S2: Information on the interaction network between protists and bacterial communities.

|  | Phototroph | Consumer | Parasite | Bacteria | Interaction | Positive | Number of communities | Keystone taxa of bacteria | Keystone taxa of protist |
| --- | --- | --- | --- | --- | --- | --- | --- | --- | --- |
| R0 | 11 | 38 | 27 | 2333 | 4119 | 54.79% | 27 | *Sphingobium fuliginis* | *Eimeria necatrix* (parasite) |
| R1 | 10 | 40 | 26 | 3448 | 7420 | 99.62% | 31 | *Xanthomonas citri* | *Rostrostelium ellipticum* (consumer) |
| R2 | 15 | 45 | 23 | 2353 | 3882 | 68.73% | 38 | *Pedobacter ginsengisoli* | *Saprolegnia diclina* (parasite) |
| R3 | 10 | 48 | 29 | 4040 | 8866 | 99.84% | 33 | *Tomitella gaofuii* | *Plasmodium coatneyi* (parasite) |
| R4 | 12 | 35 | 20 | 4313 | 1831 | 68.50% | 31 | *Xanthomonas vesicatoria* | *Phytophthora parasitica* (parasite) |

Table S3: The abundance of resistance genes within each sample.

| Sample | TCS | ARG | Cu^2+^ |
| --- | --- | --- | --- |
| S1 | 448.9094 | 478.2817 | 518.656 |
| S2 | 535.065 | 463.8491 | 512.367 |
| S3 | 423.7225 | 388.3304 | 430.268 |
| S4 | 592.3793 | 519.8141 | 710.431 |
| S5 | 564.5035 | 533.38 | 711.525 |
| S6 | 457.3491 | 494.4065 | 697.365 |
| S7 | 370.3748 | 256.0987 | 256.715 |
| S8 | 385.0711 | 307.7026 | 248.637 |
| S9 | 395.9599 | 304.1704 | 269.13 |
| S10 | 404.3594 | 573.0871 | 690.704 |
| S11 | 506.7786 | 549.1225 | 706.433 |
| S12 | 451.1957 | 480.6924 | 665.799 |
| S13 | 551.949 | 861.8203 | 44.3791 |
| S14 | 527.4312 | 965.8602 | 40.8981 |
| S15 | 541.2252 | 878.4507 | 35.6501 |

Reference

1. Gan, Y., et al., *Response of aerobic granular sludge under acute inhibition by polystyrene microplastics: Activity, aggregation performance, and microbial analysis.* Environmental Pollution, 2024. **349**: p. 123923.

2. Newton, D.P., P.-Y. Ho, and K.C. Huang, *Modulation of antibiotic effects on microbial communities by resource competition.* Nature Communications, 2023. **14**(1): p. 2398.

3. Li, L.-J., et al., *Characterizing potential pathogens from intracellular bacterial community of protists in wastewater treatment plants.* Environment International, 2023. **171**: p. 107723.

4. Shi, Y., et al., *Optimization of moving bed biofilm reactors for oil sands process-affected water treatment: The effect of HRT and ammonia concentrations.* Sci. Total Environ., 2017. **598**: p. 690-696.

5. Cabrero, A., et al., *Effects of copper and zinc on the activated sludge bacteria growth kinetics.* Water Research, 1998. **32**(5): p. 1355-1362.

6. Li, S., et al., *Long-term effect of different Cu(II) concentrations on the performance, microbial enzymatic activity and microbial community of sequencing batch reactor.* Environmental Pollution, 2019. **255**: p. 113216.

7. Zhou, J., M.A. Bruns, and J.M. Tiedje, *DNA recovery from soils of diverse composition.* Applied and Environmental Microbiology, 1996. **62**(2): p. 316-322.

8. Bolger, A.M., M. Lohse, and B. Usadel, *Trimmomatic: a flexible trimmer for Illumina sequence data.* Bioinformatics, 2014. **30**(15): p. 2114-20.

9. Li, D., et al., *MEGAHIT: an ultra-fast single-node solution for large and complex metagenomics assembly via succinct de Bruijn graph.* Bioinformatics, 2015. **31**(10): p. 1674-6.

10. Uritskiy, G.V., J. DiRuggiero, and J. Taylor, *MetaWRAP—a flexible pipeline for genome-resolved metagenomic data analysis.* Microbiome, 2018. **6**(1): p. 158.

11. Lu, J., et al., *Metagenome analysis using the Kraken software suite.* Nature Protocols, 2022. **17**(12): p. 2815-2839.

12. Li, Y., et al., *Primary succession changes the composition and functioning of the protist community on mine tailings, especially phototrophic protists.* ACS Environmental Au, 2022. **2**(5): p. 396-408.

13. Geisen, S., et al., *Soil protists: a fertile frontier in soil biology research.* FEMS Microbiology Reviews, 2018. **42**(3): p. 293-323.

14. Menzel, P., K.L. Ng, and A. Krogh, *Fast and sensitive taxonomic classification for metagenomics with Kaiju.* Nature Communications, 2016. **7**(1): p. 11257.

15. Levy Karin, E., M. Mirdita, and J. Söding, *MetaEuk-sensitive, high-throughput gene discovery, and annotation for large-scale eukaryotic metagenomics.* Microbiome, 2020. **8**(1): p. 48.

16. Suzek, B.E., et al., *UniRef clusters: a comprehensive and scalable alternative for improving sequence similarity searches.* Bioinformatics (Oxford, England), 2015. **31**(6): p. 926-932.

17. Kanehisa, M., Y. Sato, and K. Morishima, *BlastKOALA and GhostKOALA: KEGG Tools for Functional Characterization of Genome and Metagenome Sequences.* Journal of molecular biology, 2016. **428**(4): p. 726-731.

18. Kanehisa, M. and S. Goto, *KEGG: kyoto encyclopedia of genes and genomes.* Nucleic acids research, 2000. **28**(1): p. 27-30.

19. Zhang, Z.-F., et al., *Long-read assembled metagenomic approaches improve our understanding on metabolic potentials of microbial community in mangrove sediments.* Microbiome, 2023. **11**(1): p. 188.

20. Hyatt, D., et al., *Prodigal: prokaryotic gene recognition and translation initiation site identification.* BMC Bioinformatics, 2010. **11**(1): p. 119.

21. Patro, R., et al., *Salmon provides fast and bias-aware quantification of transcript expression.* Nat Methods, 2017. **14**(4): p. 417-419.

22. Yin, X., et al., *ARGs-OAP v2.0 with an expanded SARG database and Hidden Markov Models for enhancement characterization and quantification of antibiotic resistance genes in environmental metagenomes.* Bioinformatics, 2018. **34**(13): p. 2263-2270.

23. Buchfink, B., C. Xie, and D.H. Huson, *Fast and sensitive protein alignment using DIAMOND.* Nature Methods, 2015. **12**(1): p. 59-60.

24. Pal, C., et al., *BacMet: antibacterial biocide and metal resistance genes database.* Nucleic Acids Research, 2013. **42**(D1): p. D737-D743.

25. Gu, Z., *Complex heatmap visualization.* iMeta, 2022. **1**(3): p. e43.

26. Wang, Z., et al., *Environmental stress promotes the persistence of facultative bacterial symbionts in amoebae.* Ecology and Evolution, 2023. **13**(3): p. e9899.

27. Balcom, I.N., et al., *Metagenomic analysis of an ecological wastewater treatment plant's microbial communities and their potential to metabolize pharmaceuticals.* F1000Res, 2016. **5**: p. 1881.
